# C_3_ plant isotopic variability in a boreal mixed woodland: implications for bison and other herbivores

**DOI:** 10.7717/peerj.12167

**Published:** 2021-09-23

**Authors:** Jessica Z. Metcalfe

**Affiliations:** Department of Anthropology, Lakehead University, Thunder Bay, Ontario, Canada

**Keywords:** Stable isotopes, Carbon isotope, Nitrogen isotope, Terrestrial, Plant, Bison, Boreal, Isotopic baseline, Mammoth, Grass

## Abstract

Plant isotopic baselines are critical for accurately reconstructing ancient diets and environments and for using stable isotopes to monitor ecosystem conservation. This study examines the stable carbon and nitrogen isotope compositions (*δ*^13^C, *δ*^15^N) of terrestrial C_3_ plants in Elk Island National Park (EINP), Alberta, Canada, with a focus on plants consumed by grazers. EINP is located in a boreal mixed woodland ecozone close to the transition area between historic wood and plains bison habitats, and is currently home to separate herds of wood and plains bison. For this study, 165 C_3_ plant samples (grasses, sedges, forbs, shrubs, and horsetail) were collected from three habitat types (open, closed, and wet) during two seasons (summer and fall). There were no statistically significant differences in the *δ*^13^C or *δ*^15^N values of grasses, sedges, shrubs and forbs. On the other hand, plant *δ*^13^C and *δ*^15^N values varied among habitats and plant parts, and the values increased from summer to fall. These results have several implications for interpreting herbivore tissue isotopic compositions: (1) consuming different proportions of grasses, sedges, shrubs, and forbs might not result in isotopic niche partitioning, (2) feeding in different microhabitats or selecting different parts of the same types of plants could result in isotopic niche partitioning, and (3) seasonal isotopic changes in herbivore tissues could reflect seasonal isotopic changes in dietary plants rather than (or in addition to) changes in animal diet or physiology. In addition, the positively skewed plant *δ*^15^N distributions highlight the need for researchers to carefully evaluate the characteristics of their distributions prior to reporting data (*e.g*., means, standard deviations) or applying statistical models (*e.g*., parametric tests that assume normality). Overall, this study reiterates the importance of accessing ecosystem-specific isotopic baselines for addressing research questions in archaeology, paleontology, and ecology.

## Introduction

The significant difference between the stable carbon isotope compositions (*δ*^13^C) of C_3_ *versus* C_4_ plants is the foundation for many paleodiet, foodweb, and conservation studies. However, terrestrial plants utilizing C_4_ photosynthesis are rare in cool high-latitude environments, including most of Canada, Europe, and northern Asia (Lüttge, 2004; Osborne et al., 2014; Still et al., 2003). During cold intervals such as the Last Glacial Maximum, C_3_-dominated environments extended to even lower latitudes (Cotton et al., 2016). Despite the lack of C_4_ plants, animal isotopic niche partitioning can still occur within C_3_-dominated areas because of predictable variations in C_3_ plants in response to factors such as aridity, soil salinity, degree of canopy cover, carbon source (atmospheric or aquatic), nitrogen source, and mycorrhizal associations. For example, terrestrial herbivores across Pleistocene Eurasia and North America occupied different isotopic dietary niches which varied temporally and geographically (*e.g*., Bocherens, 2015; Bocherens et al., 2015; Fox-Dobbs, Leonard & Koch, 2008; Metcalfe, Longstaffe & Hodgins, 2013; Metcalfe et al., 2016; Schwartz-Narbonne et al., 2019). Isotopic niche partitioning has also been demonstrated among modern terrestrial herbivores in C_3_-dominated environments (*e.g*., Ben-David, Shochat & Adams, 2001; Cerling, Hart & Hart, 2004; Feranec, 2007; MacFadden & Higgins, 2004; Stewart et al., 2003; Urton & Hobson, 2005). Interpreting the underlying causes of animal niche partitioning requires an understanding of local baseline isotopic variations (Casey & Post, 2011).

Processes underlying variations in *δ*^13^C values of terrestrial plants utilizing the C_3_ photosynthetic pathway have been reviewed elsewhere and are described only briefly here. Terrestrial C_3_ plants have *δ*^13^C values ranging from about −37 to −20‰ when standardized to a atmospheric CO_2_ *δ*^13^C of −8.0‰ (Kohn, 2010). Environmental factors known to affect C_3_ plant *δ*^13^C values include the isotopic composition and concentration of utilized CO_2_, sources of CO_2_ (atmospheric *vs*. aquatic, ancient *vs*. modern), water availability and plant water-use efficiency, soil salinity, degree of canopy cover, and plant type/taxa (*e.g*., Hare et al., 2018; Lajtha & Michener, 1994; Tieszen, 1991). Different parts of the same plant (*e.g*., photosynthetic vs non-photosynthetic tissues) can have widely disparate *δ*^13^C values as a result of different formation times, biochemical compositions, fractionations during transportation of biomolecules within the plant, and height within the forest canopy (Cernusak et al., 2009; Chevillat et al., 2005; Ghashghaie & Badeck, 2014). Seasonal changes in plant *δ*^13^C can occur due to differing environmental conditions during growth and/or changes during maturation (*e.g*., Lowdon & Dyck, 1974; Vogado et al., 2020). Variable isotopic compositions at the base of the food chain can be passed on to herbivores with differential feeding strategies (Casey & Post, 2011). For example, caribou/reindeer tend to have high δ^13^C values relative to co-existing herbivores because of their reliance on high-^13^C lichen, and animals that feed in closed-canopy areas have lower δ^13^C values than those that feed in open areas (*e.g*., Barnett, 1994; Drucker et al., 2010).

Nitrogen isotopic variability in plants results from utilization of different molecular forms of nitrogen, manner of nitrogen uptake (*e.g*., particular mycorrhizal associations) location of nitrogen assimilation, and mobilization of nitrogen within the plant (Craine et al., 2009; Hobbie & Hogberg, 2012). Temperature, aridity, mycorrhizal type, and degree of nitrogen cycling within an ecosystem have been shown to affect plant *δ*^15^N (see Szpak, 2014 for review). Aquatic *versus* terrestrial growth can also systematically affect *δ*^15^N values (Plint, Longstaffe & Zazula, 2019). Individual plant *δ*^15^N can change over time due to a range of factors, including growth stage, seasonal conditions, soil nitrogen conditions, and decomposition (Karlsson, Eckstein & Weih, 2000; Szpak et al., 2012; Tahmasebi et al., 2017). Variations in nitrogen isotopic compositions at the base of the food chain can be passed on to consumers, leading to significant variability in *δ*^15^N even among animals feeding at the same trophic level (Casey & Post, 2011). For example, differences in the *δ*^15^N of various members of the beaver family (*Castoridae*) likely reflect differing reliance on aquatic *versus* terrestrial woody plants (Plint et al., 2020; Plint, Longstaffe & Zazula, 2019, and the high δ^15^N values of mammoths (*Mammuthus* spp.) can be attributed to selection of high-^15^N grasses (Bocherens, 2003; Metcalfe, Longstaffe & Hodgins, 2013; Schwartz-Narbonne et al., 2015).

Plant isotopic baselines for archaeological and ecological studies are crucial for interpreting the isotopic compositions of ancient humans and animals. Failure to understand or account for variations at the base of the food chain can lead to incorrect interpretations of diet, trophic level, and environmental conditions, particularly when comparing among regions or time periods (Casey & Post, 2011). However, obtaining appropriate plant isotopic baselines for a region or time period of interest can be difficult. Published surveys of modern plant natural isotopic variability are relatively rare, and the majority of those that do exist report only means, standard deviations, and data visualizations rather than a full list of the measured isotopic compositions of individual plants (Table 1). Furthermore, compilations of regional or global plant isotopic data could obscure systematic variations that occur on a local level (see discussion in Drucker et al., 2010). Thus, ecosystem-specific baselines are ideal. Ancient plants are rarely preserved except in rare depositional environments (dry caves, permafrost) or as charred remains of cooking activities (*e.g*., Metcalfe & Mead, 2019; Styring et al., 2013; Szpak & Chiou, 2019; Wooller et al., 2007), which means that archaeological and paleontological studies must rely at least in part on insights from modern plants. This is certainly true in boreal environments, where highly acidic soils often cause complete degradation of organic remains (Gordon & Buikstra, 1981; Woywitka, 2016).

**Table 1:** Studies of modern C_3_ plant natural isotopic variability in cold-temperate and boreal locations.

Environment (s)	Location	Plant life-forms	Isotopes	Full data?	Reference
Alpine meadow and steppe	Tibetan plateau	Grass, sedge, shrub, sub-shrub, forb	N	No	(Zhou et al., 2016)
Alpine meadow and steppe	Tibetan plateau	Grass, sedge, shrub, sub-shrub, forb	C	No	(Zhou et al., 2013)
Arctic	Alaska (Central & North)	Sedge, shrub, forb, lichen, moss	N	No	(Nadelhoffer et al., 1996)
Arctic/Boreal	Alaska (Interior)	Grass, sedge, horsetail, herb	C, N	No	(Funck et al., 2020)
Arctic/Boreal	Alaska (north-central)	Shrub/tree, aquatic	C, N	No	(Kielland, 2001)
Arctic/Boreal	Alaska & Yukon	Shrub/tree, herb, aquatic, fungus, lichen	C, N	No	(Ben-David, Shochat & Adams, 2001)
Arctic/Boreal	Alaska & Yukon	Grass, sedge	C	Yes	(Wooller et al., 2007)
Arctic/Boreal	Alaska (Seward Pen.)	Graminoid, shrub, forb, lichen	N	No	(Finstad & Kielland, 2011)
Arctic/Taiga	Alaska (taiga)	Shrub/tree	C, N	No	(Kielland & Bryant, 1998)
Arctic/Tundra	Alaska (North Slope)	Graminoid, forb, shrub, lichen	C, N	Yes	(Barnett, 1994)
Arctic/Tundra	Banks Island, NWT	Shrub, forb, grass, sedge, lichen, moss	C, N	Yes	(Munizzi, 2017)
Boreal forest	N. Saskatchewan	Tree, shrub, forb, moss, lichen	C	No	(Brooks et al., 1997)
Boreal Grasslands	S. Yukon	Shrub, herb	C, N	Yes	(Tahmasebi et al., 2017)
Cold-temperate/boreal forests	North America & Eurasia	Tree	C, N	No	(Kloeppel et al., 1998)
Coniferous forests	Oregon	Tree	C	No	(Bowling et al., 2002)
Grasslands, fens, *Salix* snowbeds	Greenland	Graminoid, shrub	C, N	No	(Kristensen et al., 2011)
Plains Grasslands	South Dakota & Mongolia	Tree, shrub, forb, grass	C	No	(Tieszen, 1994)
Shrubland, meadow, steppe, desert	Tibetan plateau	Graminoid, tree, shrubs, xeromorph	C	No	(Song et al., 2008)
Steppe	Inner Mongolia	Grass, shrub, sub-shrub, forb	C	No	(Chen et al., 2005)
Subarctic woodlands/shrublands	Sweden (north)	Graminoid, woody, cryptogam	N	No	(Karlsson, Eckstein & Weih, 2000)
Temperate forest	Switzerland	Tree	C	No	(Chevillat et al., 2005)
Temperate grasslands/woodlands	UK	Grass	C, N	No	(Bonafini et al., 2013)
Temperate to sub-Mediterranean	Germany & France	Tree	C, O	No	(Keitel et al., 2006)
Various	N. Scandinavia	Tree, shrub, dwarf shrub	C	No	(Loader & Rundgren, 2006)
Various	Canada (south to north)	Grass, tree, fern	C	Yes	(Lowdon & Dyck, 1974)
Wetlands	Yukon & S. Ontario	Tree, shrub, aquatic	C, N	Yes	(Plint, Longstaffe & Zazula, 2019)

Boreal mixed woodlands are important regions for understanding animal ecology and human-animal interactions. In particular, the plains-parkland transition in northern Alberta (Canada) was a critical area for both human and animal migrations, beginning with the opening of the so-called Ice-Free Corridor and continuing throughout the Late Holocene (*e.g*., Heintzman et al., 2016; Ives, 2003; Shapiro et al., 2004). Northern Alberta is home to a diverse mammalian fauna including ungulates such as moose, elk, and deer. Until the late 19^th^ century, the region was also home to abundant bison, and was an area of transition between wood bison (*Bison bison athabascae*) territory in the north (*i.e*., boreal forests of northern Alberta and Saskatchewan, the Northwest Territories, Yukon, and Alaska) and plains bison (*Bison bison bison*) territory in the south (*i.e*., the prairies and plains) (Van Zyll de Jong, 1986). The current research was motivated by a desire to use stable isotope analysis to better understand modern and archaeological/paleontological bison dietary selectivity in C_3_-dominated boreal regions, where bison have access to a range of plants and habitats. As a first step, this study examines natural variations in the carbon and nitrogen isotope compositions of plants in Elk Island National Park (EINP), Alberta, with a focus on plants that may have been consumed by bison.

### Study location: Elk Island National Park, Alberta

**Figure 1:**
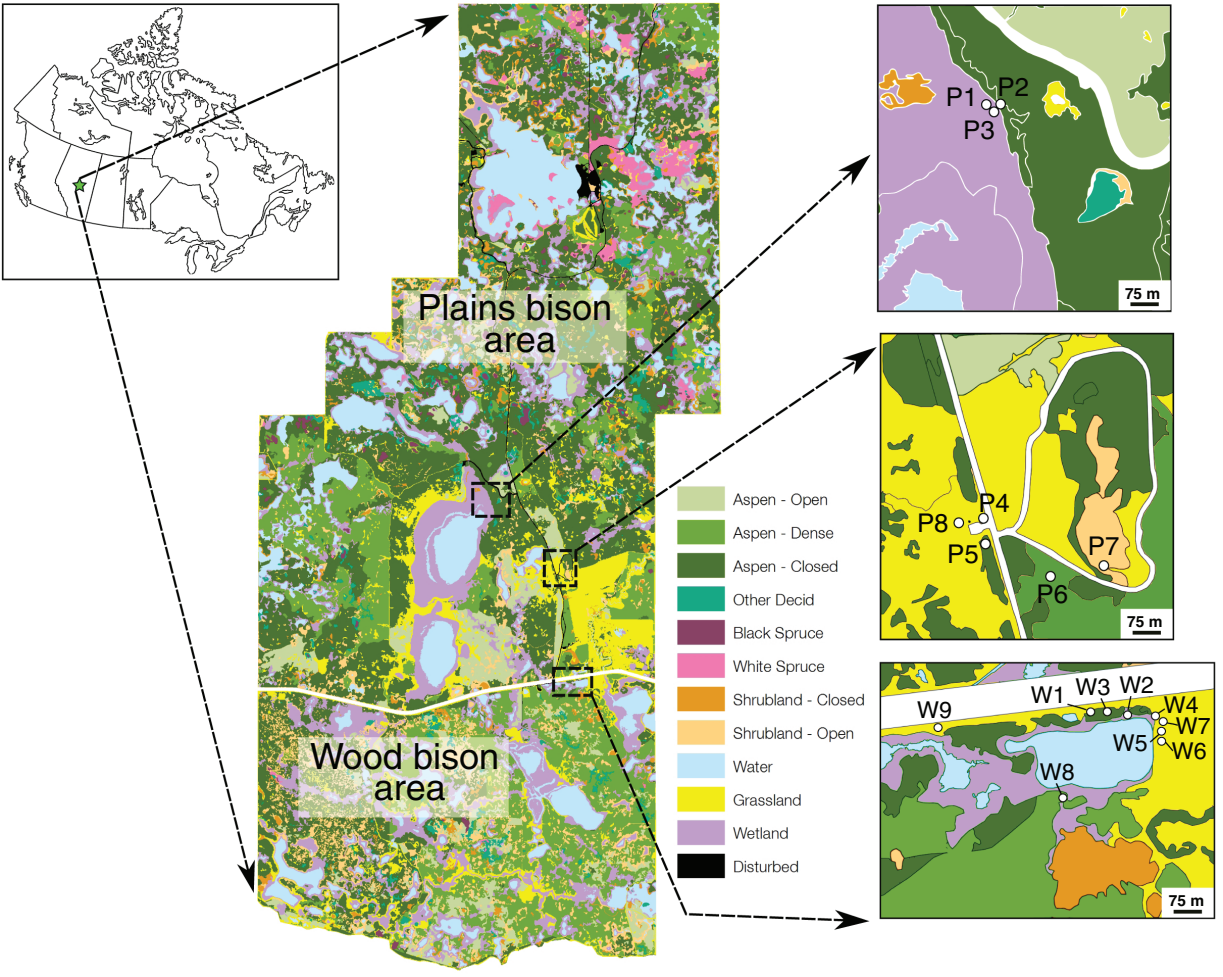
Location of Elk Island National Park and plant sampling locations relative to vegetation zones defined in a previous Parks Canada survey. During our sample collection, P1 and P3 were open and dry (not wet), whereas P7 was a small wetland (not shrubland). Other vegetation zones for sampling locations agreed with field observations during sampling. Map credit: Parks Canada, OGL.

Elk Island National Park (EINP) is a ~200 km^2^ protected area located ~40 km east of Edmonton. The park is located within Canada’s southern boreal plains ecozone, an area of transition between semi-arid prairie and wetter boreal forest (Nicholson, 1995). Topographically, the park is part of the Beaver Hills region, an area of knob-and-kettle terrain with abundant lakes and wetlands. Vegetation within the park is a patchy mosaic of aspen parkland, boreal mixed woodland, grassy/shrub meadows, marshes, and lacustrine areas (Fig. 1) (Best & Bork, 2004; Holsworth, 1960; Nicholson, 1995). All identified plant taxa in the park utilize C_3_ photosynthesis (Hanna Schoenberg, personal communication, May 18, 2021). EINP’s mean annual temperature was 1.7 °C and mean annual precipitation was 460 mm between 1951 and 1980, but both temperature and precipitation have been increasing due to climate change (https://climatedata.ca/). EINP typically experiences moderate summers and cold, dry, windy winters. Temperatures range from average lows of −18 °C in January to average highs of 23 °C in July (weather-atlas.com). Peak summer rains occur in July (mean of 112 mm precipitation) and snowfall reaches a high of 206 mm in March (weather-atlas.com). Spring blooms typically begin to appear in May and the growing season lasts from approximately mid-May to mid-September.

EINP is home to several large ungulate species, including moose (*Alces alces*), elk/wapiti (*Cervus canadensis*), white-tailed deer (*Odocoileus virginianus*), mule deer (*Odocoileus hemionus*), plains bison (*Bison bison bison*), and wood bison (*Bison bison athabascae*) (Telfer & Cairns, 1986). For many decades, EINP has been a source for genetically-pure disease-free bison that have been introduced to conservation herds across the continent (Markewicz, 2017). The plains and wood bison areas are separate: plains bison range freely within the fully-fenced northern portion of the park and wood bison range freely within the separate, fully-fenced southern portion of the park (Fig. 1). Bison in both areas have access to the same types of habitat and vegetation.

## Materials & Methods

### Sample collection and preparation

**Figure 2:**
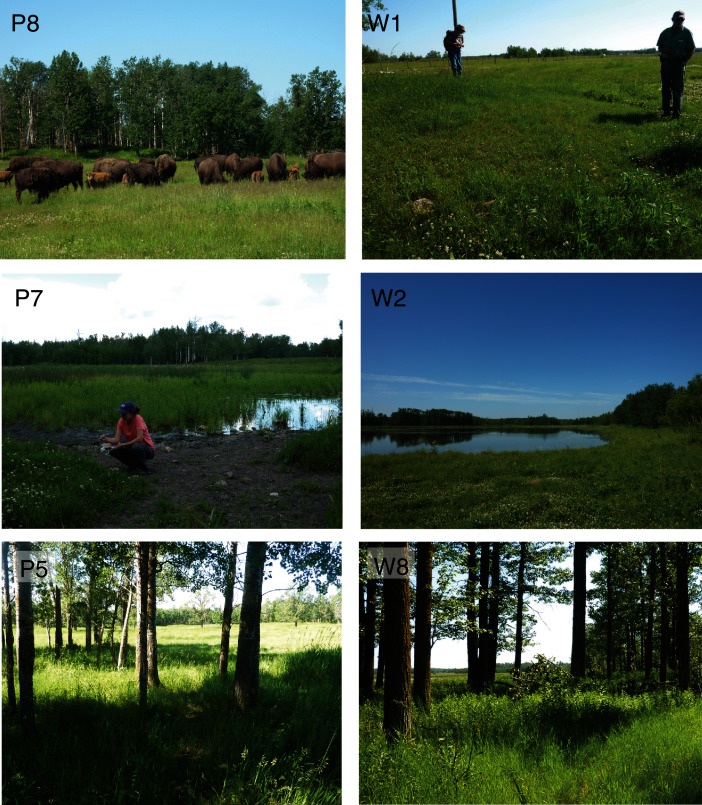
Selected plant sampling locations in the plains bison (P) and wood bison (W) sections of Elk Island National Park, including open (P8, W1), wet (P7, W2) and closed (P5, W8) areas.

Plant samples were collected with the permission of Parks Canada (Research and Collection Permit EI-2016-21863). Grass, sedge, forb, shrub, and horsetail (*Equisetum* spp.) samples were collected on June 27–29, 2016 (*n* = 133) and November 6, 2016 (*n* = 32) from dry open areas (*e.g*., meadows, hill slopes), dry closed-canopy environments, and wet areas (shorelines of ponds or lakes) (Figs. 1, 2). Site categorizations were based on observations at the time of sampling rather than on generalized vegetation maps, because wetlands can be ephemeral. Sampling sites were selected based on recent sightings of bison and physical evidence of bison (*e.g*., dung, wallows, hoofprints) in the area. To mimic bison foraging patterns, only terrestrial above-ground plant parts were collected. For the same reason, graminoids were prioritized for collection and sampled relative to their abundance at each location. Since only two sampling sites were wetlands and sedge cover was sparse, the sample size for sedges is low. Plants were identified to genus or species with reference to Johnson et al. (1995).

All samples were air-dried and ground to a fine powder with a Wig-L-Bug device prior to isotopic analysis. Most of the samples (*n* = 131) were homogenized into ‘whole plant’ samples such as might be consumed by a relatively indiscriminate herbivore, including varying proportions of leaves, stems, seeds, and/or flowers (Table 2). To test for isotopic differences among plant parts, leaves and seeds/flowers were analyzed separately for a subset of samples (*n* = 34). Grass leaves are wrapped around stems before diverging as a separate blade, making stems and leaves difficult if not impossible to separate in bulk samples. Grass flowers are complex structures that include a rachis and many tiny pedicels which are likewise difficult or impossible to separate from the floret. As a result, grass leaf and seed/flower samples include variable proportions of these other tissues as well.

**Table 2:** Elk Island National Park plant data. An Excel version of this table is available as a Supplemental File.

Site	Habitat	Season	Sample	Taxon	Type	Parts*	*δ*^13^C	*δ*^15^N	%C	%N	C/N
P1	Open	Summer	P1–10a	*Ranunculus sp*.	forb	St, L, F	−28.0	−1.0	40.4	1.3	37
P1	Open	Summer	P1–11a	*Trifolium hybridium*	forb	St, L	−30.6	−1.3	37.9	2.6	17
P1	Open	Summer	P1–12a	*Fragaria virginiana*	forb	St, L	−28.2	−2.0	39.6	1.8	26
P1	Open	Summer	P1–13a	*Poaceae*	grass	St, L	−28.4	−1.2	38.5	1.4	32
P1	Open	Summer	P1–14a	*Poa pratensis*	grass	St, Sd	−26.3		39.0		
P1	Open	Summer	P1–15a	*Poaceae*	grass	St, L, Sd	−26.3	−1.8	40.3	1.6	30
P1	Open	Summer	P1–1a	*Poa pratensis*	grass	St, Sd	−27.7		38.8		
P1	Open	Summer	P1–1b	*Poa pratensis*	grass	L	−29.0	−2.6	38.9	2.1	22
P1	Open	Summer	P1–2a	*Hedysarum alpinum*	forb	St, L, F	−28.3	−0.1	40.1	2.9	16
P1	Open	Summer	P1–3a	*Rubus pubescens*	shrub	St, L	−27.9	−1.5	40.6	2.3	20
P1	Open	Summer	P1–4a	*Phleum pratense*	grass	St, L, Sd	−28.4	−2.6	40.4	1.4	34
P1	Open	Summer	P1–5a	*Salix sp*.	shrub	L	−30.0	0.0	37.7	2.0	22
P1	Open	Summer	P1–6a	*Amelanchier alnifolia*	shrub	St, L, Fr	−25.9	−2.2	43.9	1.2	42
P1	Open	Summer	P1–7a	*Ranunculus sp*.	forb	St, L, F	−28.0	−0.7	40.4	1.7	28
P1	Open	Summer	P1–8a	*Galium boreale*	forb	St, L	−28.6	−0.7	40.3	1.6	29
P1	Open	Summer	P1–9a	*Apocynum androsaemifolium*	forb	St, L, F	−29.3	+1.6	42.4	1.8	28
P2	Closed	Summer	P2–1a	*Agrimonia striata*	forb	St, L	−30.4	−2.3	37.8	1.8	24
P2	Closed	Summer	P2–2a	*Salix sp*.	shrub	L	−28.7	+1.6	41.1	2.0	25
P2	Closed	Summer	P2–3a	*Lonicera involucrata*	shrub	L	−29.9	−1.2	38.5	1.6	28
P2	Closed	Summer	P2–4a	*Poaceae*	grass	St, L	−30.3	−0.5	36.4	1.4	31
P2	Closed	Summer	P2–5a	*Poaceae*	grass	St, L	−29.4	+1.5	36.9	1.3	33
P2	Closed	Summer	P2–6a	*Thalictrum sp*.	forb	St, L	−30.5	+1.9	39.6	2.1	22
P2	Closed	Summer	P2–7a	*Sanicula marilandica*	forb	St, F	−30.5	+0.7	38.8	2.3	20
P2	Closed	Summer	P2–7b	*Sanicula marilandica*	forb	L	−32.3	−0.1	38.6	3.2	14
P2	Closed	Summer	P2–8a	*Poa pratensis*	grass	St, L, Sd	−31.0	+1.1	36.6	1.6	27
P3	Open	Summer	P3–1a	*Poaceae*	grass	L	−27.8	+1.6	38.2	2.1	22
P3	Open	Summer	P3–1b	*Poaceae*	grass	Sd	−26.9	+1.6	39.7	2.2	21
P3	Open	Summer	P3–2a	*Poaceae*	grass	St, L	−27.8	+1.3	35.9	1.8	23
P3	Open	Summer	P3–3a	*Trifolium sp*.	forb	St, L, F	−28.4	−0.4	37.0	3.0	15
P3	Open	Summer	P3–4a	*Astragalus sp*.	forb	St, L	−27.3	+0.7	38.1	4.8	9
P3	Open	Summer	P3–5a	*Cyperaceae*	sedge	L	−27.4	+2.1	38.2	1.7	26
P3	Open	Summer	P3–5b	*Cyperaceae*	sedge	Sd	−26.6	+1.7	39.2	1.9	24
P4	Open	Summer	P4–1a	*Poaceae*	grass	Sd	−27.6	−1.3	39.7	2.2	21
P4	Open	Summer	P4–1b	*Poaceae*	grass	L	−28.5	−3.1	36.2	2.0	21
P4	Open	Summer	P4–2a	*Bromus sp*.	grass	St, Sd	−27.3	−3.2	39.2	1.4	32
P4	Open	Summer	P4–3a	*Poa pratensis*	grass	St, Sd	−25.7	−0.7	38.6	1.1	42
P4	Open	Summer	P4–3b	*Poa pratensis*	grass	St, L	−28.7	−3.6	37.4	1.0	43
P4	Open	Summer	P4–4a	*Poaceae*	grass	St, L	−28.3	−0.4	38.5	2.2	20
P4	Open	Summer	P4–6a	*Bromus sp*.	grass	Sd	−26.2	+0.1	40.5	1.8	26
P4	Open	Summer	P4–6b	*Bromus sp*.	grass	L	−28.0	−1.4	39.6	1.9	24
P4	Open	Summer	P4–7a	*Trifolium hybridium*	forb	St, L	−30.0	−1.5	35.7	2.5	17
P4	Open	Summer	P4–7b	*Trifolium hybridium*	forb	St, F	−28.2	−0.6	38.0	1.9	23
P5	Closed	Summer	P5–1a	*Poa pratensis*	grass	St, L, Sd	−29.6		37.0		
P5	Closed	Summer	P5–2a	*Poaceae*	grass	St, L	−30.3	−2.3	38.3	1.3	34
P5	Closed	Summer	P5–3a	*Bromus inermis*	grass	Sd	−29.4	−0.1	40.2	1.9	24
P5	Closed	Summer	P5–3b	*Bromus inermis*	grass	L	−30.4	−1.2	38.9	1.3	35
P5	Closed	Summer	P5–4a	*Agropyron sp*.	grass	Sd	−31.2	−2.0	39.5	1.7	28
P5	Closed	Summer	P5–4b	*Agropyron sp*.	grass	St, L	−32.2	−2.1	37.6	1.6	27
P5	Closed	Summer	P5–5a	*Phleum pratense*	grass	Sd	−29.5	+8.4	39.4	1.9	25
P5	Closed	Summer	P5–5b	*Phleum pratense*	grass	L	−31.1	+6.8	39.0	2.2	21
P5	Closed	Summer	P5–5c	*Phleum pratense*	grass	St	−29.1	+6.5	38.4	0.9	51
P5	Closed	Summer	P5–6a	*Trifolium sp*.	forb	St, L	−28.7	−2.0	34.5	2.5	16
P5	Closed	Summer	P5–7a	*Unidentified forb*	forb	St, L	−32.1	−1.7	37.9	1.6	27
P5	Closed	Summer	P5–8a	*Poaceae*	grass	L	−29.9	−1.3	39.0	2.3	20
P5	Closed	Summer	P5–9a	*Phalaris arundinacea*	grass	Sd	−28.8	+0.7	39.4	2.1	21
P5	Closed	Summer	P5–9b	*Phalaris arundinacea*	grass	L	−30.6	−0.8	37.6	2.6	17
P5	Closed	Fall	P5N–1	*Poaceae*	grass	St, L	−29.9		37.1		
P5	Closed	Fall	P5N–2a	*Poa sp*.	grass	Sd	−29.2	−1.7	39.7	1.9	24
P5	Closed	Fall	P5N–2b	*Poa sp*.	grass	St, L	−29.3		39.2		
P5	Closed	Fall	P5N–3b	*Poaceae*	grass	St, L	−29.3	+0.2	39.7	1.6	28
P5	Closed	Fall	P5N–4a	*Poa sp*.	grass	Sd	−29.9	+4.2	39.7	1.1	41
P5	Closed	Fall	P5N–4b	*Poa sp*.	grass	St, L	−29.7		39.9		
P5	Closed	Fall	P5N–5a	*Poaceae*	grass	L	−31.6		40.3		
P5	Closed	Fall	P5N–5b	*Poa sp*.	grass	Sd	−30.3	+6.7	38.9	0.8	60
P6	Open	Summer	P6–1a	*Agropyron sp*.	grass	St, L, Sd	−28.0	+1.6	39	1.7	27
P6	Open	Summer	P6–1b	*Agropyron sp*.	grass	St, L	−28.3	+1.2	37.8	3.5	12
P6	Open	Summer	P6–2a	*Bromus inermis*	grass	Sd	−27.2	+1.1	39.8	1.8	26
P6	Open	Summer	P6–2b	*Bromus inermis*	grass	St, L	−28.2	+0.3	38.5	1.9	23
P6	Open	Summer	P6–3a	*Trifolium sp*.	forb	St, L	−29.1	−2.0	37.8	3.1	14
P6	Open	Summer	P6–3b	*Trifolium sp*.	forb	St, F	−27.6	−1.3	39.1	2.3	20
P6	Open	Summer	P6–4a	*Poa pratensis*	grass	St, Sd, L	−26.9		39.5		
P6	Open	Summer	P6–5a	*Poaceae*	grass	St, L	−28.3	+1.9	38.8	1.1	42
P6	Open	Summer	P6–6	*Poaceae*	grass	L	−28.6	−2.0	38.2	4.0	11
P6	Open	Summer	P6–7a	*Poaceae*	grass	L	−28.8	+2.4	38.7	2.2	20
P6	Open	Summer	P6–8a	*Ranunculus sp*.	forb	St, L, F	−27.9	+0.8	37.0	1.7	26
P7	Wet	Summer	P7–1a	*Beckmannia syzigachne*	grass	St, L, Sd	−29.0		37.7		
P7	Wet	Summer	P7–2a	*Poa pratensis*	grass	St, L, Sd	−26.8		36.7		
P7	Wet	Summer	P7–2b	*Poa pratensis*	grass	St, L	−30.2	+2.3	37.7	2.8	16
P7	Wet	Summer	P7–3a	*Bromus inermis*	grass	St, L, Sd	−26.5	−0.1	39.8	1.6	29
P7	Wet	Summer	P7–4a	*Alopecurus aequalis*	grass	St, L, Sd	−28.4	+6.5	38.4	1.6	29
P7	Wet	Summer	P7–4b	*Alopecurus aequalis*	grass	L	−29.1	+6.5	38.1	2.5	18
P7	Wet	Summer	P7–5a	*Calamagrostis canadensis*	grass	Sd	−28.1	+2.9	38.7	1.9	24
P7	Wet	Summer	P7–5b	*Calamagrostis canadensis*	grass	L	−30.0	+2.0	35.3	1.9	22
P7	Wet	Summer	P7–6a	*Poaceae*	grass	St, L	−30.2	+4.8	37.1	3.0	14
P7	Wet	Summer	P7–7a	*Agropyron trachycaulum*	grass	St, L, Sd	−27.2	+2.1	38.3	2.3	19
P8	Open	Summer	P8–1a	*Poaceae*	grass	St, L	−28.8	+0.9	37.0	1.8	24
P8	Open	Summer	P8–2a	*Poa pratensis*	grass	St, L, Sd	−25.5	−0.6	37.7	0.8	57
P8	Open	Summer	P8–2b	*Poa pratensis*	grass	L	−28.6	+1.6	37.5	2.0	22
P8	Open	Summer	P8–3a	*Poaceae*	grass	St, Sd	−27.0	+0.1	38.2	0.9	50
P8	Open	Summer	P8–3b	*Poaceae*	grass	L	−28.7	+2.2	37.5	2.2	20
P8	Open	Summer	P8–3c	*Poaceae*	grass	L	−28.9	+1.6	37.6	2.0	22
P8	Open	Summer	P8–3d	*Agropyron trachycaulum*	grass	St, Sd	−27.5	+2.7	37.8	1.5	29
P8	Open	Fall	P8N–1a	*Poa sp*.	grass	Sd	−27.2	−1.9	39.2	1.1	40
P8	Open	Fall	P8N–1b	*Poaceae*	grass	St, L	−27.8	+1.1	39.2	0.9	52
P8	Open	Fall	P8N–1c	*Agropyron trachycaulum*	grass	Sd	−26.4	+1.0	39.1	0.9	51
P8	Open	Fall	P8N–1d	*Agropyron trachycaulum*	grass	St	−26.5		41.3		
P8	Open	Fall	P8N–2a	*Agropyron trachycaulum*	grass	Sd	−25.6	+9.9	41.2	1.2	42
P8	Open	Fall	P8N–2b	*Agropyron trachycaulum*	grass	St	−25.4		42.8		
P8	Open	Fall	P8N–2c	*Poaceae*	grass	L	−28.7	+7.5	38.3	0.7	65
P8	Open	Fall	P8N–3a	*Poa sp*.	grass	Sd	−28.5	−0.6	38.0	0.9	48
P8	Open	Fall	P8N–3b	*Poa sp*.	grass	St	−27.5		40.3		
P8	Open	Fall	P8N–3c	*Poaceae*	grass	L	−29.0	+1.8	35.5	0.6	66
P8	Open	Fall	P8N–4a	*Agropyron trachycaulum*	grass	Sd	−27.1	+4.3	41.1	1.0	48
P8	Open	Fall	P8N–4b	*Agropyron trachycaulum*	grass	St	−27.9		41.4		
P8	Open	Fall	P8N–4c	*Poaceae*	grass	St, L	−28.5	+3.1	39.8	1.2	39
P8	Open	Fall	P8N–5a	*Poaceae*	grass	L	−28.7		40.1		
W1	Open	Summer	W1–1a	*Poaceae*	grass	St, L	−30.4	−3.2	37.4	1.0	43
W1	Open	Summer	W1–2a	*Poa sp*.	grass	St, L, Sd	−28.3	−2.7	37.3	1.0	42
W1	Open	Summer	W1–3a	*Bromus sp*.	grass	St, L, Sd	−27.2	−3.9	38.2	0.9	51
W1	Open	Summer	W1–3b	*Bromus sp*.	grass	Sd	−28.3	−1.6	38.7	1.7	27
W1	Open	Summer	W1–4a	*Poa pratensis*	grass	St, L	−29.9	−3.8	37.6	1.3	33
W1	Open	Summer	W1–4b	*Poa pratensis*	grass	St, Sd	−28.8	−3.5	38.2	1.2	37
W1	Open	Summer	W1–5a	*Phleum pratense*	grass	WS	−30.8	−1.6	38.2	0.8	54
W2	Wet	Summer	W2–1a	*Poaceae*	grass	St, L	−30.1	+0.4	38.2	1.8	25
W2	Wet	Summer	W2–2a	*Poa sp*.	grass	St, L	−30.5	−1.5	36.6	1.6	26
W3	Closed	Summer	W3–1a	*Poaceae*	grass	St, L	−31.1	−1.4	35.2	1.3	31
W3	Closed	Summer	W3–2a	*Poaceae*	grass	St, L	−32.6	−2.4	36.2	0.9	47
W3	Closed	Fall	W3N–1a	*Poaceae*	grass	St, L	−29.5		39.8		
W3	Closed	Fall	W3N–2a	*Poaceae*	grass	L	−29.3		39.6		
W3	Closed	Fall	W3N–3a	*Poaceae*	grass	L	−30.2	+0.7	41.0	0.8	61
W4	Open	Summer	W4–1a	*Carex atheroides*	sedge	St, L, Sd	−27.9	+0.4	37.9	1.2	38
W4	Open	Summer	W4–2a	*Carex sp*.	sedge	L	−29.8	+2.9	37.1	1.4	31
W4	Open	Summer	W4–3a	*Poaceae*	grass	St, L	−29.7	−1.2	38.8	1.6	28
W4	Open	Summer	W4–3b	*Poaceae*	grass	St, L, Sd	−29.0	−1.1	37.8	1.3	35
W4	Open	Summer	W4–4a	*Carex atherodes*	sedge	St, L, Sd	−27.7	+0.9	38.7	1.3	35
W4	Open	Summer	W4–4b	*Carex atherodes*	sedge	St, L	−27.9	−0.5	37.6	1.1	39
W5	Open	Summer	W5–1a	*Poa sp*.	grass	St, Sd	−26.9	−1.0	38.4	1.0	46
W5	Open	Summer	W5–1b	*Poa sp*.	grass	L	−27.9	+0.5	38.6	2.2	20
W5	Open	Summer	W5–2a	*Agropyron sp*.	grass	St, L, Sd	−26.4	+1.7	38.7	2.1	22
W5	Open	Summer	W5–2b	*Agropyron sp*.	grass	L	−28.8	+1.2	37.7	2.4	18
W5	Open	Summer	W5–3a	*Poaceae*	grass	St, L	−28.2	+2.5	38.2	3.6	12
W6	Open	Summer	W6–1a	*Phleum pratense*	grass	St, L, Sd	−29.2	−0.5	37.9	1.4	32
W6	Open	Summer	W6–1b	*Phleum pratense*	grass	L	−26.6	+2.9	38.5	2.8	16
W6	Open	Summer	W6–2a	*Poa sp*.	grass	St, L, Sd	−29.7	−2.2	38.6	0.9	50
W6	Open	Summer	W6–2b	*Poa sp*.	grass	St, L	−30.4	−0.3	36.3	1.7	25
W7	Open	Summer	W7–1a	*Equisetum sp*.	horsetail	St, L	−29.6	+4.5	30.7	1.6	22
W7	Open	Summer	W7–2a	*Poa sp*.	grass	Sd	−27.7	−0.9	37.4	1.2	36
W7	Open	Summer	W7–2b	*Poa sp*.	grass	St, L	−28.6	−0.1	37.2	1.4	31
W7	Open	Summer	W7–3a	*Phleum pratense*	grass	Sd	−26.7	−1.2	39.5	1.5	31
W7	Open	Summer	W7–3b	*Phleum pratense*	grass	St, L	−28.1	−2.4	38.1	1.1	40
W8	Closed	Summer	W8–1a	*Agropyron sp*.	grass	St, L	−29.7	−1.9	37.9	1.5	29
W8	Closed	Summer	W8–2a	*Bromus sp*.	grass	St, Sd	−28.4	+6.5	39.7	2.1	22
W8	Closed	Summer	W8–3a	*Equisetum sp*.	horsetail	WS	−30.6	+3.2	32.7	1.7	23
W8	Closed	Summer	W8–4a	*Poaceae*	grass	L	−29.6	−3.9	37.6	0.9	47
W8	Closed	Summer	W8–5a	*Poaceae*	grass	St, L	−29.8	−0.6	38.5	2.0	23
W8	Closed	Summer	W8–6a	*Poaceae*	grass	L	−28.3	−1.0	36.9	1.1	38
W8	Closed	Summer	W8–7a	*Bromus sp*.	grass	Sd	−29.1	−0.6	40.4	2.1	23
W8	Closed	Summer	W8–7b	*Bromus sp*.	grass	St, L, Sd	−28.8	−1.4	39.6	1.6	29
W8	Closed	Summer	W8–8a	*Poaceae*	grass	St, L, Sd	−28.2		38.1		
W9	Open	Summer	W9–1a	*Poa sp*.	grass	St, Sd	−26.9		38.2		
W9	Open	Summer	W9–2a	*Poaceae*	grass	L	−28.1	−0.9	38.2	1.4	33
W9	Open	Summer	W9–3a	*Melilotus officinalis*	forb	St, L, F	−27.7	−1.2	37.6	2.4	18
W9	Open	Summer	W9–4a	*Equisetum sp*.	horsetail	WS	−29.1	+5.1	33.0	2.0	19
W9	Open	Summer	W9–5a	*Poaceae*	grass	St, L	−28.4	+0.7	38.0	1.5	31
W9	Open	Summer	W9–6a	*Bromus sp*.	grass	St, L, Sd	−25.4		39.0		
W9	Open	Summer	W9–7a	*Poaceae*	grass	St, L, Sd	−26.9	−0.3	37.9	1.4	31
W9	Open	Summer	W9–8a	*Trifolium hybridium*	forb	St, F	−26.3	−1.2	38.0	2.0	22
W9	Open	Summer	W9–8b	*Trifolium hybridium*	forb	St, L	−27.9	−1.4	36.4	3.4	13
W9	Open	Fall	W9N–1a	*Poa sp*.	grass	Sd	−26.7	−0.9	40.1	0.9	51
W9	Open	Fall	W9N–1b	*Poa sp*.	grass	St, L	−26.2		40.6		
W9	Open	Fall	W9N–2a	*Poaceae*	grass	St, L	−28.1		38.9		
W9	Open	Fall	W9N–3a	*Poaceae*	grass	Sd	−27.1	+5.7	41.9	1.0	49
W9	Open	Fall	W9N–3b	*Poaceae*	grass	St	−24.9		43.8		
W9	Open	Fall	W9N–4a	*Bromus sp*.	grass	Sd	−25.6	+2.2	40.4	1.4	35
W9	Open	Fall	W9N–4b	*Bromus sp*.	grass	St, L	−25.3		41.9		

**Notes:**

* St, stem; L, leaf; F, flower; Fr, fruit; Sd, seed; WS, whole sample.

Many grass samples lacked an inflorescence, making more specific identification difficult or impossible.

### Carbon and nitrogen isotope measurements

Carbon and nitrogen isotope values (*δ*^13^C, *δ*^15^N) and carbon and nitrogen contents (dry weight %C, %N) were obtained using an Elementar VarioMicro Cube elemental analyzer coupled with an Isoprime isotope-ratio mass spectrometer in continuous-flow mode. Carbon and nitrogen isotope values were obtained during the same run by combusting approximately 1 mg of sample and using a high level of dilution to reduce the carbon dioxide gas peaks. Nitrogen isotope results from samples with N_2_ peaks <1 nA were excluded unless duplicate analyses exhibited similar reproducibility to samples with larger gas peaks. Carbon isotope values of the low-nitrogen samples were retained since the carbon peaks were large enough to produce reliable results. The samples with low nitrogen-gas peaks are those lacking *δ*^15^N values in Table 2.

*δ*^13^C values were calibrated to VPDB and *δ*^15^N values were calibrated to AIR using USGS-40 and USGS-41 or 41a (accepted *δ*^13^C values of −26.39, +37.63 and +36.55‰ and accepted *δ*^15^N values of −4.52, +47.57 and +47.55‰, respectively). Sample replicates (minimum 10% of samples in each run) and internal check standards of methionine, amaranth, and red lentil (long-term mean *δ*^13^C of −28.60, −13.59, −26.12‰; long-term mean *δ*^15^N of −5.04, +2.94 and −1.09‰, respectively) were used to monitor measurement uncertainty. Uncertainty measures were calculated following the method of Szpak, Metcalfe & Macdonald (2017). For *δ*^13^C, precision *u(R*_*w*_) was 0.11‰, accuracy (*u(bias)*) was 0.09‰, and total analytical uncertainty (*u*_*c*_) was 0.14‰. For *δ*^15^N, precision was 0.23‰, accuracy was 0.23‰, and total analytical uncertainty was 0.33‰.

### Statistical analyses

Statistical analyses were conducted using Excel for Office 365 and PAST (PAleontological STatistics) 4.03. Shapiro–Wilk W tests were used to assess the normality of distributions. Levene tests were used to evaluate the homogeneity of variance. Normally distributed datasets (carbon isotope values) were compared using Student’s t-tests (two independent samples), paired-sample t-tests (two paired samples), or one-way ANOVA F-tests with Tukey’s post-hoc comparisons (three or more independent samples). Non-normally distributed datasets (nitrogen isotope values) were compared using Mann–Whitney U tests (two independent samples), Wilcoxon sign-rank tests (two paired samples) or Kruskal–Wallis H tests with Dunn-Bonferroni post-hoc comparisons (three or more independent samples). Alpha was set to 0.05 for all statistical comparisons. In the text below, means are reported with standard deviations, unless noted otherwise.

## Results

### Whole sample

**Figure 3:**
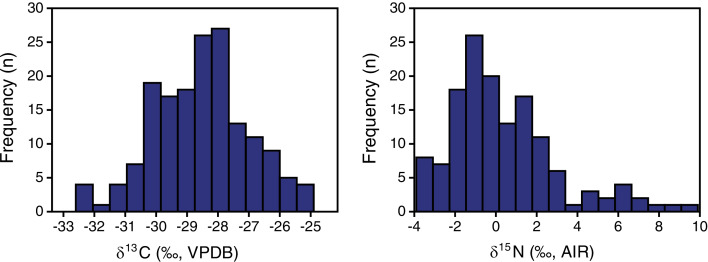
EINP plant carbon and nitrogen isotope distributions.

Plant *δ*^13^C values ranged from −32.6 to −24.9‰, with a mean and standard deviation of −28.5 ± 1.5‰ (Tables 2, 3). Plant *δ*^15^N values ranged from −3.9 to +9.9‰, with a mean and standard deviation of +0.4 ± 2.7‰. The shape of the distribution was normal for *δ*^13^C (Shapiro−Wilk W = 0.99, *n* = 165, *p* = 0.7; skewness = −0.05) and positively skewed for *δ*^15^N (Shapiro–Wilk W = 0.92, *n* = 141, *p* < 0.001; skewness = 1.14) (Fig. 3).

**Table 3:** Summary statistics for EINP plant samples grouped all together (whole sample) and by type, habitat, parts, and season of collection.

	*δ*^13^C (‰, VPDB)					*δ*^15^N (‰, AIR)				
	*n*	Mean	Median	SD	Range	*n*	Mean	Median	SD	Range
Whole Sample	165	−28.5	−28.5	1.5	−32.6 to −24.9 (7.7)	141	+0.4	−0.3	2.7	−3.9 to +9.9 (13.8)
Type										
Grass	128	−29.0	−28.5	1.5	−32.6 to −24.9 (7.7)	104	+0.5	−0.1	2.9	−3.9 to +9.9 (13.8)
Sedge	6	−27.9	−27.8	1.1	−29.8 to −26.6 (3.2)	6	+1.3	+1.3	1.2	−0.5 to +2.9 (3.4)
Shrub	5	−28.5	−28.7	1.7	−30.0 to −25.9 (4.1)	5	−0.7	−1.2	1.5	−2.2 to +1.6 (3.8)
Forb	23	−29.0	−28.4	1.5	−32.3 to −26.3 (6.0)	23	−0.7	−1.0	1.2	−2.3 to +1.9 (4.2)
Horsetail	3	−29.8	−29.6	0.8	−30.6 to −29.1 (1.5)	3	+4.3	+4.5	0.6	+3.2 to +5.1 (1.9)
Habitat										
Open	108	−27.9	−28.0	1.2	−30.8 to −24.9 (5.9)	94	+0.1	−0.4	2.4	−3.9 to +9.9 (13.8)
Closed	45	−30.0	−29.9	1.1	−32.6 to −28.2 (4.4)	37	+0.5	−0.6	3.1	−3.9 to +8.4 (12.3)
Wet	12	−28.8	−29.1	1.4	−30.5 to −26.5 (4.0)	10	+2.6	+2.2	2.7	−1.5 to +6.5 (8.0)
Parts (same plant)										
Leaf	34	−29.0	−28.8	1.5	−32.3 to −25.3 (7.0)	28	+0.5	−0.1	2.8	−3.6 to +7.5 (11.1)
Seed	34	−27.8	−27.7	1.5	−31.2 to −25.5 (5.7)	28	+1.0	0.0	3.0	−2.2 to +9.9 (12.1)
Season										
Summer (all)	133	−28.7	−28.5	1.5	−32.6 to −25.4 (7.2)	124	+0.1	−0.5	2.4	−3.9 to +8.4 (12.3)
Summer (match*)	32	−29.0	−28.9	1.9	−32.6 to −25.4 (7.2)	29	+0.5	−0.6	2.9	−2.4 to +8.4 (10.0)
Fall	32	−28.0	−28.3	1.7	−31.6 to −24.9 (6.7)	17	+2.5	+1.8	3.4	−1.9 to +9.9 (11.8)

**Note:**

* Summer (match) excludes data from locations that were not sampled in Fall.

### Plant types

**Figure 4:**
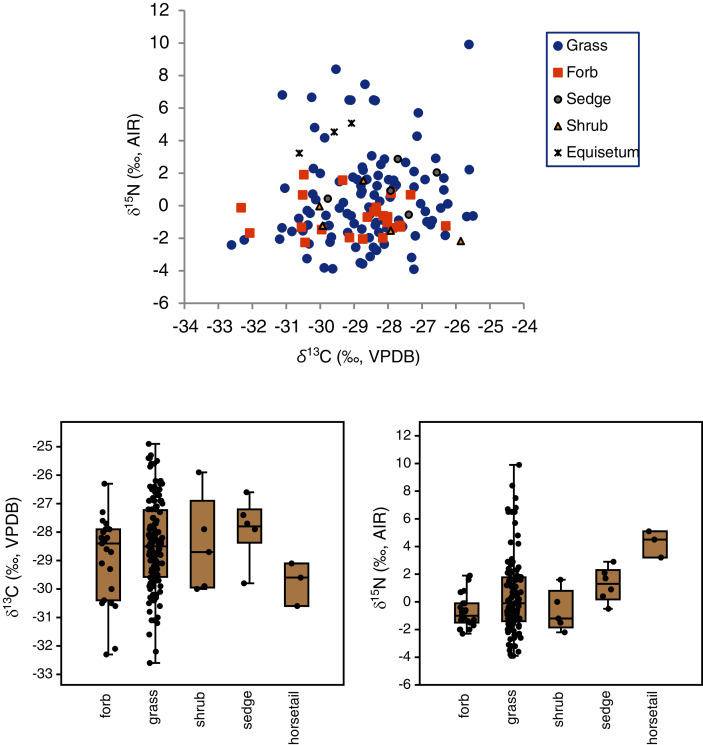
Carbon and nitrogen isotope compositions of EINP plants grouped by type.

The mean δ^13^C values of grasses, sedges, shrubs, forbs and horsetail were within 1.9‰ of one another (Table 3), and an ANOVA showed no statistically significant differences among the groups (F(4,160) = 1.3, *p* = 0.28). With horsetail removed (because of its small sample size), there were still no significant differences in δ^13^C among grasses, sedges, shrubs, and forbs (F(3,158) = 1.0, *p* = 0.39) There was a significant difference among the *δ*^15^N values of plant types (H(4) = 12.9, *p* = 0.01), but the Dunn–Bonferroni test suggested that only the horsetail-forb comparison was significant (*p* = 0.03). With horsetails removed there was no statistically significant difference among grasses, sedges, shrubs, and forbs (H(3) = 7.0, *p* = 0.07), and their medians were within 2.3‰ of one another. Although the median grass *δ*^15^N value did not significantly differ from that of any other group, grasses had the greatest variability of any plant type, and grass samples had both the highest (>+5.1‰) and lowest (<−2.3‰) individual plant *δ*^15^N values (Table 3, Fig. 4). A Levene’s test from medians (*i.e*., Brown–Forsythe test) indicated that the difference in the variability of δ^15^N among plant types was statistically significant (*p* = 0.01).

### Habitats

**Figure 5:**
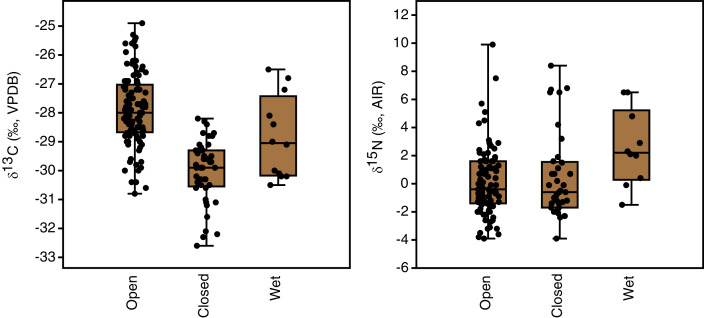
Plant carbon and nitrogen isotope distributions by growth habitat. The box encloses the interquartile range and median (horizontal line). The whiskers represent the full range of measured values.

Plant growth habitat had a significant effect on the carbon isotope compositions of plants (F(2,162) = 48.8, *p* < 0.001). The differences among all three groups were statistically significant, with the highest *δ*^13^C values in open areas (−27.9 ± 1.2‰, *n* = 108), intermediate values in wet areas (−28.9 ± 1.4‰, *n* = 12) and the lowest values in closed-canopy areas (−30.0 ± 1.1‰, *n* = 45) (Table 3, Fig. 5). Growth habitat also affected *δ*^15^N values (H(2) = 7.7, *p* = 0.02), with higher *δ*^15^N values in wet habitats (+2.6 ± 2.7‰, *n* = 10) compared to those in either open areas (+0.1 ± 2.4‰, *n* = 94) or closed canopy areas (+0.5 ± 3.1‰, *n* = 37). Although wet areas had higher mean (and median) *δ*^15^N values than the open or closed-canopy areas, the latter two habitat types hosted the plants with the highest individual *δ*^15^N measurements (Fig. 5). As mentioned previously, these extreme δ^15^N values were all from grass samples. There was a positive skew in the *δ*^15^N values of plants from open environments (W = 0.9, *n* = 94, *p* < 0.001) and closed environments (W = 0.9, *n* = 37, *p* < 0.001).

### Plant parts

**Figure 6:**
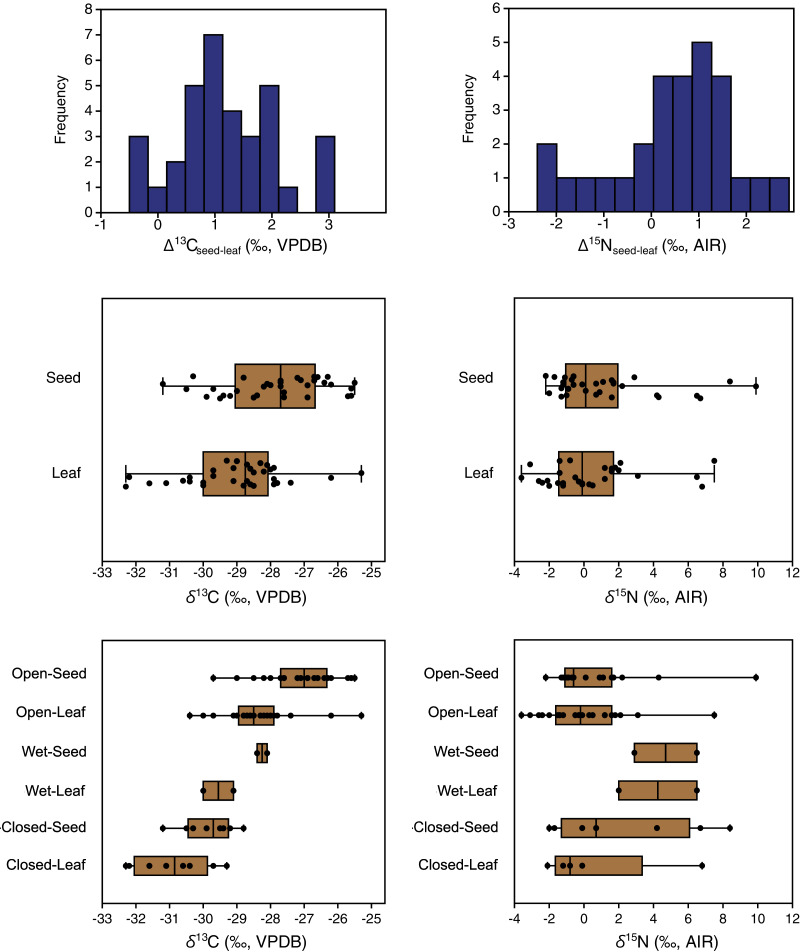
Differences between the carbon and nitrogen isotopic compositions of seeds and leaves from the same plants.

Carbon isotope compositions of leaves were on average 1.2‰ lower than those of seeds/flowers from the same plants (paired samples t = 7.8, df = 33, *p* < 0.001) (Table 3). Furthermore, the great majority of individual plant samples had lower leaf than seed/flower *δ*^13^C values, with seed/flower minus leaf differences (Δ^13^C_seed–leaf_) of individual plants ranging from −0.5 to +3.1‰ (Fig. 6). The lowest mean and individual *δ*^13^C values were obtained from leaves in closed habitats, and the highest mean *δ*^13^C from seeds in open habitats (Fig. 6).

**Figure 7:**
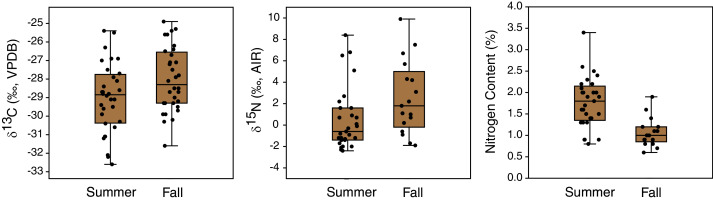
Comparison of carbon and nitrogen isotope compositions and nitrogen contents of EINP plants collected from matched locations in summer (late June) and fall (early November).

Nitrogen isotope compositions of leaves were 0.5‰ lower on average than those of seeds/flowers from the same plants (Table 3), but the difference was not statistically significant (Wilcoxon W = 250, df = 27, *p* = 0.06). Individual plants had highly variable seed-minus-leaf differences (Δ^15^N_seed–leaf_), ranging from −2.4 to +2.9‰ (Fig. 7).

### Seasonal changes

Seasonal shifts in plant *δ*^13^C and *δ*^15^N occurred between early summer (late June) and mid fall (early November) (Table 3, Fig. 7). Plant *δ*^13^C increased slightly during fall, both for the whole dataset (t(163) = 2.1, *p* = 0.04, mean difference of 0.6‰) and when only locations sampled in both seasons were included (t(62) = 2.2, p = 0.03; mean difference of 1.0‰). Plant *δ*^15^N also increased during fall, both for the whole dataset (U = 582.5, *p* = 0.003; mean difference of 2.5‰) and when only samples from matched locations were compared (U = 145, *p* = 0.02; mean difference of 2.0‰). Plant nitrogen contents (%N) also significantly decreased from summer to fall (whole sample: U = 265, *p* < 0.001; mean difference of 0.8% matched locations: U = 71, *p* < 0.001; mean difference of 0.7%) (Fig. 7). The true seasonal decrease in plant nitrogen content is likely greater than this value implies, since proportionally more fall plant samples were excluded due to their small gas peaks (see Table 3).

## Discussion

### Plant isotopic distributions

The distribution of plant *δ*^13^C values was normal. The EINP whole-sample mean *δ*^13^C of −28.5‰ is somewhat lower than the modern global mean C_3_ plant *δ*^13^C value of −27.0‰ determined by Kohn (2010). This can be attributed to two main factors: (1) the *δ*^13^C of atmospheric CO_2_ during our sample collection (in 2016) was significantly lower than Kohn’s (2010) normalized value of −8.0‰ because of the ongoing effects of fossil fuel burning (Long et al., 2005), and (2) Kohn’s (2010) study excluded understory plants with *δ*^13^C values below −31.5‰, whereas the present study did not.

Distributions of plant nitrogen isotope compositions were positively skewed. Skewness of isotopic distributions is seldom explicitly evaluated, and isotopic data presentations that facilitate visual examination of skewness (*e.g*., frequency histograms, box-and-whisker plots) are relatively rare, so it is difficult to determine how common skewed plant nitrogen isotope distributions may be. Metcalfe & Mead (2019) observed a negatively skewed *δ*^15^N distribution for Pleistocene plants. Funck et al. (2020: Supplemental Material) provide box-plots that appear to illustrate positively skewed modern grass *δ*^15^N and negatively skewed modern herb *δ*^15^N distributions, but they did not explicitly evaluate skewness. The other plant isotopic studies reviewed here neither evaluated skewness nor presented data in forms that make it easy for readers to evaluate. Determining the shape of a distribution is often overlooked but testing for normality is a critical first step before utilizing parametric statistical methods, at least when sample sizes are small (which is typical in most archaeological and paleontological studies) (Ghasemi & Zahediasl, 2012). Failing to recognize skewed isotopic distributions can result in inappropriate data reporting (*e.g*., use of means and standard deviations rather than medians and interquartile ranges) and use of statistical tests whose assumptions are not met (*i.e*., parametric statistical tests), potentially producing invalid results and leading to erroneous interpretations. Assessing the skewness of dietary components (and other characteristics of data distribution) is also critical for studies using stable isotope mixing models, which typically assume normal distributions and require dietary inputs of means and standard deviations (Cheung & Szpak, 2020).

It is possible that skewed plant *δ*^15^N distributions could help explain the strong nitrogen isotopic niche partitioning that has been observed among herbivores in some ecosystems. In particular, mammoths tend to have significantly higher *δ*^15^N values than co-existing herbivores, which is related to a dietary (rather than physiological) difference (Schwartz-Narbonne et al., 2015). In the present study, grasses had the greatest variability in *δ*^15^N of any plant taxon and all of the most positive δ^15^N values in the skewed tail of the distribution (*i.e*., values >5.1‰) were from grasses (Table 3, Fig. 4). Grasses are the predominant food of mammoths, but also of bison, who do not have enriched *δ*^15^N values. If variables could be identified that predict which grass specimens within a given ecosystem have high *δ*^15^N values (*i.e*., taxa, parts, growth-stages, growth habitats), then it might be possible to determine if mammoths were likely to have been selecting such grasses (for example, by employing different feeding strategies or preferring different microhabitats). In general, a herbivore preferentially selecting plants from the skewed ‘tail’ of an isotopic distribution would be predicted to occupy a distinct isotopic niche relative to herbivores that are randomly selecting plants from throughout the distribution. This would also be true of herbivores selecting plants whose *δ*-values fall within the tails of a normal distribution, but a skewed plant distribution would be predicted to result in greater herbivore isotopic niche differentiation due to the more extreme values of outliers in the skewed tail of the distribution.

### Plant types

The overlapping δ^13^C and δ^15^N values of grasses, sedges, forbs, and shrubs in EINP highlights the importance of understanding local plant variability when interpreting herbivore isotopic compositions. Previous research has established some generalities about isotopic differences among primary producers. For example, lichens often have higher δ^13^C values than terrestrial plants (*e.g*., Brooks et al., 1997; Teeri, 1981), woody gymnosperms generally have higher δ^13^C values than woody angiosperms (Hare & Lavergne, 2021), and aquatic plants tend to have higher δ^15^N values than terrestrial plants (*e.g*., Kielland, 2001; Plint, Longstaffe & Zazula, 2019. However, comparisons of differences among plant types at local levels can produce disparate results (*e.g*., Drucker et al., 2010: Fig. 4), which is perhaps not surprising when one considers the complex range of environmental factors that affect δ^13^C and δ^15^N, as well as the fact that researchers select different plant groups for study and even categorize them differently (Table 1). Compilations of isotopic data from plants growing in various habitats (*i.e*., global or regional datasets) can obscure the effects of microhabitats (*e.g*., degree of canopy cover, altitude, aridity, etc.), which may be more important variables for interpreting herbivore isotopic compositions than plant type. Studies that compare herbivore isotopic compositions in ancient C_3_ ecosystems to a plant baseline organized by plant type (*e.g*., Schwartz-Narbonne et al., 2019; Schwartz-Narbonne et al., 2021) presuppose that type is the most important predictor of a plant’s isotopic compositions. An alternative approach is to put equal or greater emphasis on major environmental factors that influence plant isotopic compositions, such as the canopy effect (*e.g*., Drucker et al., 2008; Hofman-Kamińska et al., 2018) or ecosystem changes (*e.g*., Drucker et al., 2011; Metcalfe & Longstaffe, 2014).

The plant-type data in the present study highlight the importance of ecosystem-specific contexts. In particular, it is not appropriate to assume that grasses, sedges, shrubs and forbs will have consistent relative isotopic differences in disparate environments and temporal intervals. Consequently, similar isotopic niches among animals does not necessarily mean that animals ate the same things, or that “one species could fulfill another’s ecological role” (Schwartz-Narbonne et al., 2019: 1). Rather, isotopic niche overlap could simply indicate that there are minimal isotopic differences among the disparate plants consumed by herbivores in that environment. Likewise, minimal isotopic variations in serially-sampled animal tissues does not necessarily suggest that animals had highly specialized diets with minimal variation. On the contrary, minimal seasonal isotopic variations in herbivore tissues could occur even when animals undertake significant seasonal changes in diet if there are no significant differences among plant types in that area. Given these complexities, the key to being able to make meaningful interpretations of herbivore isotopic compositions is to have a good understanding of which isotopic baselines and variables are most important for a particular study and to seriously consider alternative interpretations based on the various factors that can influence isotopic systems. In general, isotopic niches are far from equivalent to dietary niches or dietary specializations.

### Plant parts and habitats: carbon isotopes

Lower plant *δ*^13^C values in EINP closed habitats compared to open habitats (~2‰ on average) is consistent with the well-known canopy effect, in which understory plants have significantly lower *δ*^13^C values than plants that make up the canopy or emergent layers, or plants that grow in open areas (*e.g*., Bonafini et al., 2013; Chevillat et al., 2005; Drucker et al., 2008; Van Der Merwe & Medina, 1991). The lower *δ*^13^C values in EINP leaves relative to seeds/flowers (~1‰ on average) is likewise in agreement with the 1–3‰ difference that has been reported in many other studies (*e.g*., Badeck et al., 2005; Ghashghaie & Badeck, 2014; Metcalfe & Mead, 2019).

The EINP plant isotopic data suggest that among herbivores, a combined effect of plant-part and habitat-selection could result in significant carbon isotope niche partitioning within C_3_ environments, with the largest differences between animals consuming seedy/flowery plants in open environments (higher *δ*^13^C) and those selecting seedless/flowerless plants in closed environments (lower *δ*^13^C). This offers an alternative to assuming that animal niche partitioning in C_3_ environments is due to differing proportions of grass vs browse or consumption of different plant taxa. Many previous studies have used herbivore *δ*^13^C to infer the ‘openness’ of utilized habitats (*e.g*., Bocherens et al., 2015; Doppler et al., 2017; Drucker et al., 2003; Drucker et al., 2011), but few have considered the additional isotopic effects of plant-part differences, such as the decrease in leaf δ^13^C than occurs as the leaf expands (Vogado et al., 2020) or differences among seedier *versus* seedless plant parts (but see Guiry et al., 2020 for an exception). The effects of ‘seedy’ vegetation on herbivore isotopic compositions deserves further study, since there may also be differential digestibility among seeds and leaves that influences their incorporation into herbivore tissues.

In general, herbivore feeding specializations go beyond selection of particular plant forms, species and habitats to include specialization on particular plant parts and growth stages. These differential feeding strategies might have particularly pronounced isotopic effects in an environment like the mammoth steppe, where co-existing grazers likely consumed different parts of the same plants. For example, elephantids rip out tall (potentially seedy) bunches of grasses by grabbing them with their trunks, whereas bison break off short (probably less seedy) grasses and tall/mid-level new growth with their tongues and teeth (Guthrie, 1982). On the mammoth steppe, bison tended to have higher *δ*^13^C values than mammoths in a range of locations and temporal intervals (*e.g*., Bocherens, 2015). Higher *δ*^13^C values in a taxon that consumes shorter grasses is the opposite of what would be expected if ‘seediness’ was a factor in isotopic niche differentiation. However, the higher *δ*^13^C values of bison could result from bison consuming a larger proportion of short, newly-grown leaves, which tend to have higher δ^13^C values than older mature leaves (Vogado et al., 2020). Regardless of what drives isotopic niche differentiation on the mammoth steppe, the results of the present study suggest that in some environments, habitat and plant-part selection could have greater isotopic effects on herbivore isotopic compositions than selection of different plant taxa.

### Plant habitat: nitrogen isotopes

EINP plants from the wet habitat tended to have higher *δ*^15^N values than plants from the dry (open or closed-canopy) environments. Although this contrasts with the general trend towards higher δ^15^N values in drier locations that is often observed on regional and global scales (Craine et al., 2009; Handley et al., 1999; Wang et al., 2014), it is consistent with the higher plant *δ*^15^N values often observed in aquatic systems relative to terrestrial systems (*e.g*., Cloern, Canuel & Harris, 2002; Kielland, 2001; Plint, Longstaffe & Zazula, 2019. It is possible (and perhaps likely) that EINP terrestrial plants growing in seasonally wet areas obtained some nitrogen from aquatic sources, leading to higher *δ*^15^N values. It is also possible that herbivore dung is frequently deposited in wetland areas when animals come to drink, contributing ^15^N-enriched nitrogen to the wetland system and mimicking the established effects of manuring on plant δ^15^N (*e.g*., Bogaard et al., 2007; Szpak et al., 2014). It is important to note that the sample size available for EINP wetland habitats was small, so the reliability of this habitat difference should be re-examined in future studies. Nevertheless, in combination with previous studies that clearly show higher *δ*^15^N values among aquatic plants, these results suggest caution for archaeologists and paleoecologists who interpret higher herbivore *δ*^15^N as indicators of increased aridity. An alternative explanation (among others) for high herbivore *δ*^15^N values could be the consumption of plants growing in or near nutrient-rich wetlands.

### Seasonal changes in plant isotopic compositions

A summer-to-fall (late June to early November) increase in both *δ*^13^C and *δ*^15^N (by ~1 and 2‰, respectively) was observed in EINP plants. This could be due to a combination of factors, including changes in the biochemical compositions of tissues, changes in source C and N isotopic compositions, remobilization of nutrients into roots for winter, and early decomposition. The direction and magnitude of seasonal isotopic changes in plants may vary among environments and locations. For example, Karlsson, Eckstein & Weih (2000) found that the *δ*^15^N values of most Subarctic plants in northern Sweden increased between the snowmelt (May) and mid-June, but decreased in August and September, with a range in seasonal variation of 2.1 to 5.3‰. On the other hand, the timing of key seasonal changes (*e.g*., temperature increases and decreases) varies considerably among locations and makes seasonal generalizations challenging.

Reconstructing ecosystem-specific seasonal changes in plant δ^13^C and δ^15^N could help researchers interpret serial-sampling studies of herbivore isotopic compositions, which may vary due to seasonal changes in diet, physiology, and/or isotopic variations in plants. Seasonal changes in the diets of a range of herbivores have been studied within C_3_-dominated ecosystems, and these changes are often relatively small in magnitude (~2 to 3‰ or less). For example, Funck et al. (2020) observed temporal changes in sectioned wood bison (*Bison bison athabascae*) hair *δ*^13^C and *δ*^15^N that they attributed to nutritional stress. Julien et al. (2012) serially-sampled steppe bison (*Bison priscus*) teeth and interpreted small winter increases in *δ*^13^C as an indication of lichen consumption. Metcalfe & Longstaffe (2014) identified different seasonal patterns in the tooth enamel of mastodons (*Mammut americanum*) that lived in the same geographical area during different time periods, which they suggested were the result of major vegetational shifts. Kielland (2001) serially-sampled Alaskan moose (*Alces alces*) hooves and interpreted variations of about 2–3‰ as evidence for seasonal changes in diet. Plant isotopic values and their variability underlie the interpretations of all these studies.

Bison generally consume graminoids year-round but may seasonally switch between grasses and sedges, and/or consume forbs and woody plants when graminoids are not available (Gogan et al., 2010). The minimal isotopic differences among plant taxa in EINP suggests that these seasonal shifts in bison foraging strategies might not be recorded in the isotopic compositions of incrementally growing bison tissues such as teeth or hair. However, based on the EINP seasonal plant data, one might predict that seasonal isotopic shifts in the plants themselves could be recorded in serially-sampled bison tissues. Generalizing to other environments, researchers should be aware that seasonal changes in herbivore isotopic compositions do not necessarily indicate changes in foraging strategies, but can result from isotopic changes within the plants themselves.

## Conclusions

This study has provided a plant carbon and nitrogen isotope baseline for future studies of herbivores in Elk Island National Park, and for archaeological and paleontological studies of animals in C_3_-dominated environments. A strong positive skew to the plant nitrogen isotope distributions highlights the need for isotopic researchers to explicitly evaluate the characteristics of their distributions (*e.g*., normal *versus* skewed) so that they can select appropriate measures of central tendency and variability, conduct appropriate statistical tests, and/or utilize isotopic mixing models.

In this study no statistically significant differences were observed in the *δ*^13^C or *δ*^15^N of the majority of C_3_ plant types (grasses, sedges, forbs, and shrubs), but there were differences among plant parts, habitats, and seasons. These results carry three important implications. First, animals consuming different plant taxa could have similar or identical isotopic compositions. Second, animals consuming the same C_3_ plant taxa could have different isotopic compositions if they select plants growing in different habitats (*e.g*., open, closed, wet) and/or different plant parts (*e.g*., leaves, seeds). Third, seasonal changes in herbivore isotopic compositions need not indicate a shift in foraging strategy, but rather could result from seasonal isotopic changes within dietary plants. Based on first principles of isotope systematics, these conclusions are not new. However, too often isotopic niche partitioning is equated with dietary niche partitioning, and a lack of isotopic niche partitioning is taken to reflect similar or identical diets. It is critical that researchers bear in mind the complexities of isotopic systems when making paleodietary inferences, and support their interpretations with explicit independent lines of evidence on plants and animals (*i.e*., isotopic baselines) in relevant ecosystems and at appropriate scales of analysis.

## Supplemental Information

10.7717/peerj.12167/supp-1 Supplemental Information 1 Elk Island National Park plant data.

## Additional Information and Declarations

### Field Study Permissions

The following information was supplied relating to field study approvals (*i.e*., approving body and any reference numbers): Sample collection was approved by Parks Canada (Research and Collection Permit EI-2016-21863).

### Data Availability

The following information was supplied regarding data availability: The full dataset is available in Table 2 and the Supplemental File.
